# Pre-ejection period by radial artery tonometry supplements echo doppler findings during biventricular pacemaker optimization

**DOI:** 10.1186/1476-7120-9-20

**Published:** 2011-07-28

**Authors:** Nima Taha, Jing Zhang, Reza Rafie, Rupesh Ranjan, Salima Qamruddin, Tasneem Z Naqvi

**Affiliations:** 1 Non Invasive Diagnostic Services and Echocardiography Laboratory, Cardiovascular and Thoracic Institute, Keck School of Medicine, University of Southern California, Los Angeles, USA; 2 Beijing Anzhen Hospital, Beijing, China

**Keywords:** Radial artery tonometry, pre-ejection time, biventricular pacing, heart failure, echo Doppler

## Abstract

**Background:**

Biventricular (Biv) pacemaker echo optimization has been shown to improve cardiac output however is not routinely used due to its complexity. We investigated the role of a simple method involving computerized pre-ejection time (PEP) assessment by radial artery tonometry in guiding Biv pacemaker optimization.

**Methods:**

Blinded echo and radial artery tonometry were performed simultaneously in 37 patients, age 69.1 ± 12.8 years, left ventricular (LV) ejection fraction (EF) 33 ± 10%, during Biv pacemaker optimization. Effect of optimization on echo derived velocity time integral (VTI), ejection time (ET), myocardial performance index (MPI), radial artery tonometry derived PEP and echo-radial artery tonometry derived PEP/VTI and PEP/ET indices was evaluated.

**Results:**

Significant improvement post optimization was achieved in LV ET (286.9 ± 37.3 to 299 ± 34.6 ms, p < 0.001), LV VTI (15.9 ± 4.8 cm to 18.4 ± 5.1 cm, p < 0.001) and MPI (0.57 ± 0.2 to 0.45 ± 0.13, p < 0.001) and in PEP (246.7 ± 36.1 ms to 234.7 ± 35.5 ms, p = 0.003), PEP/ET (0.88 ± 0.21 to 0.79 ± 0.17, p < 0.001), and PEP/VTI (17.3 ± 7 to 13.78 ± 4.7, p < 0.001). The correlation between comprehensive echo Doppler and radial artery tonometry-PEP guided optimal atrioventricular delay (AVD) and optimal interventricular delay (VVD) was 0.75 (p < 0.001) and 0.69 (p < 0.001) respectively. In 29 patients with follow up assessment, New York Heart Association (NYHA) class reduced from 2.5 ± 0.8 to 2.0 ± 0.9 (p = 0.004) at 1.8 ± 1.4 months.

**Conclusion:**

An acute shortening of PEP by radial artery tonometry occurs post Biv pacemaker optimization and correlates with improvement in hemodynamics by echo Doppler and may provide a cost-efficient approach to assist with Biv pacemaker echo optimization.

## Introduction

Prolongation of the pre-ejection period (PEP) and shortening of ejection time (ET) is the principal abnormality in systolic heart failure (HF) patients with reduced left ventricular ejection fraction (LVEF) [1]. PEP is comprised of electromechanical delay (EMD) and isovolumic contraction time(IVCT). PEP/LVET is a validated measurement of LV systolic performance and is independent of heart rate [2,3]. Myocardial performance index (MPI) is a combined measure of systolic and diastolic dysfunction [4], which indicates the non-effective fraction and has been widely used to estimate LV function and predict the prognosis in HF patients [5].

Cardiac resynchronization therapy (CRT) also referred to as atrial-synchronous biventricular (Biv) pacing, has been an effective treatment for medically refractory HF patients with ventricular dyssynchrony [1,6]. It is able to shorten PEP, lengthen ET and reduce MPI thus leading to an increase in LV effective fraction and stoke volume [6,7]., Thus PEP, ET and MPI have been regarded as the primary parameters to guide pacemaker optimization [8,9]. Maximum hemodynamic improvement occurs at an AV delay (AVD) that provides the most favorable preload and an interventricular delay (VVD) that exhibits minimal dyssynchrony, the longest ET and the shortest PEP for a dilated and failing heart [10-14].

In current practice, despite careful selection, as many as 30-40% of patients do not respond to, or even deteriorate after CRT [15]. An important reason for such high non-responder rate is lack of individual patient pacemaker optimization. Echocardiography has been the most recognized method for pacemaker optimization and is endorsed by recent guidelines from American Society of Echocardiography [10] and European Society of Cardiology [11]. It has been shown to improve cardiac output, [10,14,18] reduce hospitalization rates [16] and improve New York Heart Association (NYHA) class [17] in HF patients. Measurement of IVCT is not feasible by echo [18] and MPI measurement which requires simultaneous assessment of mitral inflow and aortic outflow is difficult to obtain during online evaluation. LV outflow velocity time integral (VTI) measurement is more reliable, however difficult to obtain due to changes in sample volume positioning. Assessment of multiple Doppler parameters such as LV filling and ejection as well as other Doppler parameters makes the procedure more reliable however also makes it more time and labor intensive. Furthermore it requires advanced skill in echo Doppler and need for multispecialty collaboration leading to underutilization of echo Doppler for pacemaker optimization. Thus methods that are simple and accurate for assisting echo Doppler techniques or better still be usable as stand-alone methods may lead to a wider adoption of pacemaker optimization.

We have recently shown the utility of phonocardiographic S3 intensity in guiding pacemaker optimization, however the technique appears to work best in those with baseline S3 of > 5 [19]. The maximal d*P*/d*t *of the radial pulse appears to be a valuable and reproducible peripheral criterion of LV systolic performance in heart failure [20]. In recent studies we have demonstrated the potential value of ejection time measured by radial artery tonometry in patients undergoing Biv pacemaker optimization [21]. Recent enhancement with a wrist-band tonometer instead of a hand held pen-like device allows a hand-free operation and capture of radial artery waveform without the variability associated with the examiner hand motion. In addition the device now allows an easy and quick assessment of PEP through computerized analysis of a characteristic point ("foot of the wave" or "timing point") on the transcutaneous radial artery waveform and the ECG-Q wave averaged approximately 10 cardiac cycles. This value comprises of two components, PEP of aortic waveform and the travel time of the pressure wave from heart to radial artery that remains constant in each patient.

We hypothesized that PEP by radial artery tonometry may correspond with ET and VTI by echo and may be used during Biv pacemaker optimization. This study was designed to investigate the relationship of PEP measurement by radial artery tonometry with echo derived ET and LV VTI, during Biv pacemaker optimization.

## Methods

### Subjects

This study protocol was approved by the institutional review board, and all subjects signed a written informed consent before enrollment. The study group comprised of 37 patients who were referred to our center for Biv pacemaker optimization by their physicians from several practices within a 50 mile radius. All patients had sinus rhythm except 4 patients with atrial fibrillation. 21 of these patients were also included in another optimization and have been reported as part of the study group in an earlier publication [19]. Patients were referred to our tertiary care facility post CRT, mainly due to lack of response from CRT, initial response to CRT followed by recurrence of symptoms and rarely for worsening of symptoms post CRT.

### Study Protocol

New York Heart Association (NYHA) class was assessed at baseline. Patient medication list was entered. The pacemaker was interrogated using the algorithm reported previously [19,21]. Underlying cardiac anatomy and physiology were reviewed by echocardiogram in the left lateral decubitus position before the procedure. Blinded echocardiographic and radial artery tonometry evaluation was performed simultaneously at baseline and during pacemaker optimization. Two independent experts performed tailored-echo and radial artery tonometry-PEP guided pacemaker optimization. Investigators performing offline analysis were blinded to online assessment of optimal AVD and VVD settings. Pacemaker was programmed based on the results of the online echocardiographic evaluation at the end of procedure.

### Tailored-Echo Guided Optimization

Examination was performed using a GE vivid 7 ultrasound system (GE Vingmed Ultrasound, Horten, Norway), a variable frequency phased array and low frequency (2.5 MHz) transducer with conventional methods [22,23]. A highest quality ECG signal was displayed on the ultrasound system and 5 cardiac cycles were used for each data acquisition for patients in sinus rhythm. For patients in atrial fibrillation or those with atrial or ventricular ectopics, 10 beats were acquired in each view. PW Doppler sample volume was placed 0.5-1 cm below the aortic valve to obtain the LV ET and VTI. Frame rate was kept above 100 fps by using single focus, narrow imaging sector, appropriate depth and frame rate. The heart rate was kept constant during optimization. Parallel Doppler beam alignment to myocardial segments and color Doppler was used for all Doppler data acquisition. Raw data was stored digitally as DICOM cine loops and transferred for offline analysis to a customized dedicated workstation equipped with custom built software (Echo PAC PC Dimension version 6.0.1) via internet.

Cardiac chamber dimensions were obtained in end systole in the parasternal long axis view. LVEF was measured by the biplane Simpson's method [23]. LV VTI, ET, diastolic filling time (FT) and MPI were measured from pulsed wave Doppler. PEP was also measured by echo as time interval from the onset of QRS complex on the ultrasound monitor and onset of aortic ejection on PW Doppler. Mitral regurgitation (MR) was graded semi-quantitatively as mitral regurgitation jet area in relation to left atrial area [24] and averaged in apical four, two and three chamber views. Pulmonary artery peak systolic pressure (PAP) was measured by continuous wave Doppler from tricuspid regurgitation jet [25].

AVD optimization was performed first and tested in the available range of 30-300 ms. Ritter's [26] method was used if feasible. In patients with mitral valve closure occurring before pacemaker spike at the long AVD, Ritter method was not used. AVD was changed in increments of 10-20 ms depending on native AVD and mitral inflow pattern [14]. PW Doppler of LV outflow tract was performed and LV ejection duration and peak velocity was measured at each AV and VVD. Optimal AVD was selected based on the "best diastolic LV filling pattern" and highest LV ejection duration and peak velocity, however in patients who had diastolic MR or tricuspid regurgitation, significant systolic MR, restrictive pulmonary vein filling pattern, prominent pulmonary vein atrial reversal and measureable and elevated PAP, these parameters were re-evaluated at optimal AVD to ensure maximum improvement in diastolic MR or tricuspid regurgitation, least restrictive pulmonary vein filling pattern (least S:D reversal, highest D wave deceleration time and minimum pulmonary vein atrial reversal), least PAP and minimum systolic MR along with optimum LV VTI and diastolic filling pattern. In some patients mitral filling time (FT) had to be compromised to minimize pulmonary vein flow reversal while in others mitral inflow A wave had to be truncated to avoid diastolic MR. LV VTI, MPI and mitral inflow VTI were measured offline.

Optimal AV delay was programmed, and then VV delay adjusted at the optimal AV delay. During VVD optimization progressively increasing LV pre-excitation was tested from 5 ms to 20 ms and right ventricular pre-excitation tested from 5 ms to 20 ms. Progressively increasing LV or right ventricular pre-excitation was tested until maximum LV ejection duration was obtained. In patients with mild or more MR and measurable PAP, these were re-evaluated to ensure minimum values at optimal VVD. Mitral inflow was re-evaluated after VVD testing and AVD readjusted if required. Overall the pacemaker setting was programmed to the AVD and VVD combination exhibiting the maximum LVET, VTI, optimum FT, minimum mitral regurgitation and pulmonary artery pressure. This was called tailored-echo guided "optimal setting".

### Radial artery tonometry-PEP Measurement

Radial artery pressure waveforms were acquired using a high-fidelity pressure transducer (Millar Instruments, Houston, TX) on the patient's wrist by an elastic band and connected to a radial artery tonometry pulse wave velocity measurement system with a simultaneously acquired 3-leads ECG signal using SphygmoCor^® ^device (AtCor Medical Inc, Lisle, IL). The sensor cartridge used in the wrist strap tonometer is the same as that used in the standard AtCor device. The only difference is that the sensor cartridge is attached to an elastic strap instead of incased in the plastic tube. Earlier comparison studies within AtCor between the standard and wrist strap tonometers showed no performance differences. These were bench tests to confirm the sensitivity and frequency response of the tonometer.

The radial artery waveform and ECG signals were then digitized and analyzed using a custom software program which determined the time interval from the ECG-Q wave to the foot (onset) of the corresponding radial artery waveform. To position the pressure transducer, the radial artery in each arm was palpated and the wrist with the stronger pulse was used. A pillow was placed under the arm to stabilize the wrist and the transducer was placed against the skin at the location where the operator felt the strongest signal from the pulse. The strap of tonometer was adjusted to capture a signal of adequate strength. The transducer was then left in place without having to be held by the operator and only needed minor adjustments in position during the data acquisition.

Examination was carried out simultaneously with the echocardiographic evaluation beginning 5-10 beats following each pacemaker adjustment. The computer screen displayed a continuous radial artery waveform and lead-II ECG wave. When 10-seconds of consistent and high quality waveforms were displayed, a computer keystroke captured and analyzed the recordings. Approximately 10 Q- radial artery foot time intervals were averaged to produce a mean value of the PEP for each record. Quality-control information was reported simultaneously to ensure consistent quality of the recordings and obtain reliable results. PEP/ET and PEP/VTI indices were calculated based on the radial artery tonometry and echo data. The optimal pacemaker setting by radial artery tonometry was taken as the AVD and VVD with the shortest PEP.

At the end of the optimization procedure the pacemaker was programmed based on the results of the online echocardiographic evaluation. NYHA class was assessed at baseline and at 1 month post optimization.

### Intraobserver and Interobserver Variability of PEP

Radial artery tonometry automated PEP measurements from different AVDs were randomly selected (every 3rd AVD observation) and a total of 46 PEP observations from 5 randomly selected patients were measured for intraobserver PEP variability, calculated as the difference of each observation from the averagex100. Interobserver variability was determined by difference of PEP from an average of 5 readings at baseline among 2 observers in 5 subjects. The interobserver variability of PEP was 2.2 ± 2.3% and intraobserver variability was 1 ± 1.6%.

### Statistical Analysis

Data are presented as mean values ± standard deviation (SD). Continuous variables at baseline and post optimization were compared using paired two-tailed student's t-test. Wilcoxon signed-rank test was used for ordinally scaled parameters like NYHA and mitral regurgitation severity. Pearson correlation analyses were performed between PEP and ET and VTI and between tailored-echo and radial artery tonometry-PEP methods for optimal AVD and VVD. Values of p < 0.05 was considered statistically significant.

## Results

Clinical characteristics of patients at baseline are shown in Table 1. All patients had > 85% Biv pacing at baseline. Patients had significant cardiac enlargement and poor LV function. 37 (97%) had symptoms of HF and 19 (51.5%) had NYHA class III-IV at baseline. Satisfactory signals by radial artery tonometry device were obtained in all patients on first attempt. In one patient with hypotension, more time was required to obtain good quality radial artery waveform.

**Table 1:** Clinical Characteristics of Study Subjects at Baseline (N = 37)

**Age**		69.1 ± 12.8 years
**Gender**	Male/Female	26(70%)/11(30%)
**NYHA**	I/II/III/IV	2.5 ± 0.8 2(5%)/16(43%)/14(38%)/5(14%)
**Rhythm**	Sinus/Atrial Fibrillation	33(89%)/4(11%)
**Etiology**	Ischemic/Non- Ischemic	25(68%)/12(32%)
**QRS Duration**		176 ± 19 ms
**Other Clinical** **Conditions**	Hypertension Diabetes Mellitus	22(66%) 12(36%)
**Medications**	Diuretics Beta-blockers ACE-inhibitors Amiodarone	28(75%) 29(78%) 26(70%) 13(35%)
**Echocardiographic** **Parameters**	Left Ventricular Diastolic Diameter Left Ventricular Systolic Diameter Left Ventricular EDV(4C) Left Ventricular ESV(4C) Left Ventricular ESV(2C) Left Ventricular EDV(2C) Left Ventricular Ejection Fraction Left Atrial Diameter (Antero-Posterior) Left Atrial Volume Left Atrial volume (Indexed)	6.3 ± 0.98 cm 5.3 ± 0.94 cm 218 ± 124 ml 153 ± 99 ml 224 ± 129 ml 156 ± 109 ml 33 ± 10% 5 ± 1 cm 108 ± 56 ml 53 ± 23 ml/m^2^

ACE = Angiotensin converting enzyme inhibitor; EDV, end diastolic volume; ESV, end systolic volume

The shortest AVD and intrinsic AVD tested in the study subjects were 66.6 ± 35.2 ms and 239.4 ± 53.7 ms respectively during pacemaker interrogation. Baseline pacemaker settings were AVD of 160 ± 53.7 ms and LV offset of 8.4 ± 14.4 ms and optimal pacemaker settings were AVD of 150.4 ± 67.2 ms and LV offset of 3.9 ± 6.7 ms (p = NS vs. baseline). A significant individual variation was present in the change in AVD setting in each patient ranging from -180 ms to +150 ms AVD from baseline. Baseline LV offset was programmed in 14 patients and RV offset in 2 patients. LV offset was added in 5 patients and removed from 7 patients and RV offset was added in 3 patients and removed in 1 patient. VVD was 0 ms post optimization in 21 patients.

**Figure 1:**
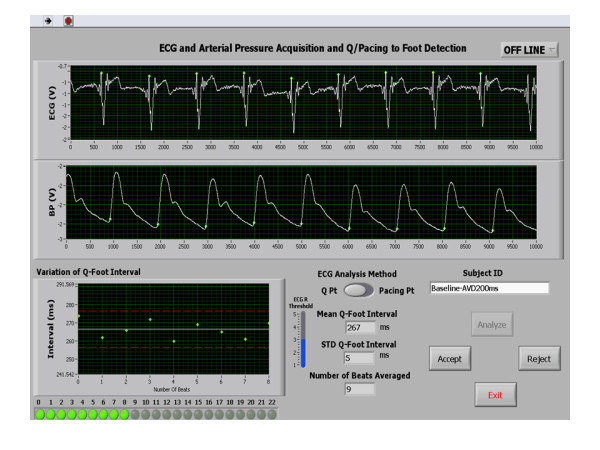
**A Case Example Showing Radial Artery PEP Measurement at Baseline**. A case example of a patient showing radial artery-PEP measurement at baseline.

**Figure 2:**
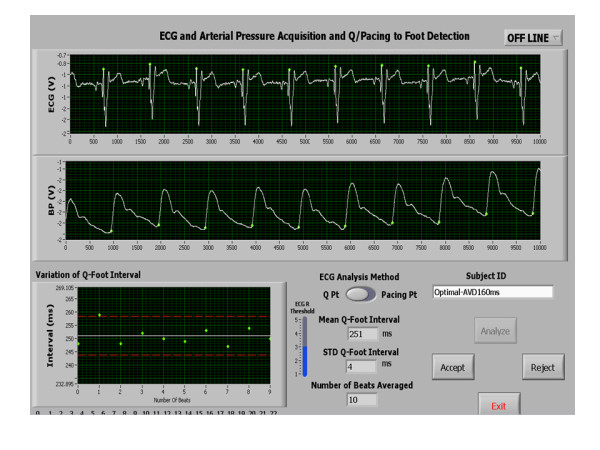
**A Case Example showing Radial Artery PEP Measurement After Echo-Guided Pacemaker Optimization**. Radial artery tonometry PEP measurement at optimal AVD in the same patient showing shortening of PEP compared to baseline. PEP, pre-ejection period.

**Figure 3:**
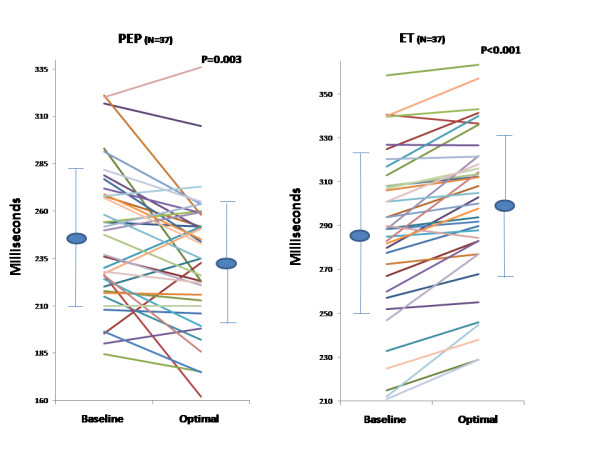
**Effect of Biventricular Pacemaker Optimization on PEP Measured by Radial Artery Tonometry and LV ET Measured by Echo Doppler**. Effect of biventricular pacemaker optimization on PEP measured by radial artery tonometry and LV ET measured by echo Doppler. Baseline and optimal PEP (left panel) and baseline and optimal ET measured by echo Doppler (right panel) are shown in each study subject. PEP, pre-ejection period; ET, ejection time

**Figure 4:**
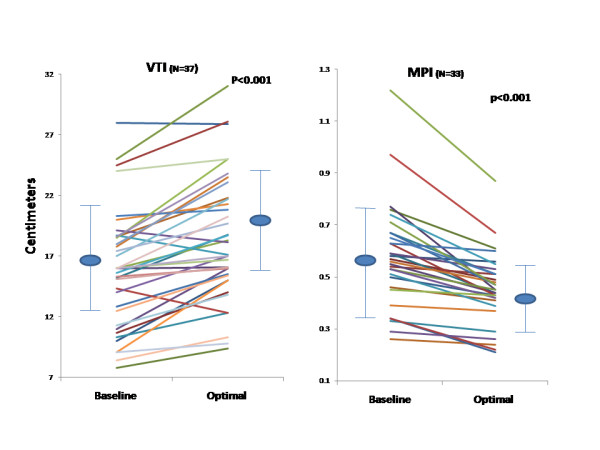
**Effect of Biventricular Pacemaker Optimization on VTI and MPI Measured by Echo Doppler**. Effect of biventricular pacemaker optimization on LV VTI and MPI measured by echo Doppler. Baseline and optimal LV VTI (left panel) and baseline and optimal MPI (right panel) measured by echo are shown in each study subject. VTI, velocity time integral; MPI, myocardial performance index. MPI was feasible in 33 patients due to atrial fibrillation in 4 patients.

An acute significant improvement was achieved post optimization in echo Doppler and radial artery tonometry measurements as shown in Table 2. Figures 1 and 2 are representative PEP recordings in a study patient at baseline and optimal AVD respectively. Data on each patient for radial artery tonometry derived PEP and echo Doppler LVET is shown in Figure 3 and on LV VTI and MPI by echo Doppler in each study subject is shown in Figure 4.

**Table 2:** Effect of Tailored Echo Guided Biventricular Pacemaker Optimization

Parameter	Baseline	Post Optimization	Percent Change	P value
**SBP (mm Hg)**	110 ± 22			
**DBP (mm Hg)**	62 ± 10			
**HR (bpm)**	66 ± 7	67 ± 7	1 ± 7%	0.6
**Echocardiographic**				
**MR (grade)**	1.5 ± 1.1	1.1 ± 0.93	-16.1 ± 26%	0.001
**PAP (mmHg)**	46 ± 10.8	38 ± 10.4	-18.5 ± 11.8%	< 0.001
**LV VTI (cm)**	15.9 ± 4.8	18.4 ± 5	17.4 ± 16%	< 0.001
**ET (ms)**	286.9 ± 37.3	299 ± 34.6	4.5 ± 4%	< 0.001
**FT (ms)**	422.5 ± 96.2	435.2 ± 81.4	4.8 ± 13.2%	0.12
**E/A ratio**	1.8 ± 1.3	1.6 ± 1.3	4.8 ± 24.3%	0.094
**MI VTI(cm)**	19.3 ± 8.4	19.7 ± 8.8	1.97 ± 14.9%	0.473
**MPI**	0.57 ± 0.2	0.45 ± 0.13	-19.5 ± 10.8%	< 0.001
**PEP(ms)**	136 ± 36	131.6 ± 40	-2.6 ± 17.6	0.24
**Radial Artery Tonometry**				
**PEP (ms)**	246.7 ± 36.1	234.7 ± 35.5	-4.5 ± 9.5%	0.003
**Radial Artery Tonometry and Echocardiographic**				
**PEP/ET**	0.88 ± 0.21	0.79 ± 0.16	-8.5 ± 9.5%	< 0.001
**PEP/VTI**	17.3 ± 7	13.7 ± 4.7	-17.5 ± 13.5%	< 0.001

MR = Mitral Regurgitation; PAP = Pulmonary Artery Pressure; LV VTI = Left Ventricular Outflow Velocity Time Integral; FT = LV Filling Time; ET = LV Ejection Time; PEP = Pre-ejection Period; MPI = Myocardial Performance Index; MR Grade: 0 = None; 0.5 = Trace; 1 = Mild; 1.5 = Mild to Moderate; 2 = Moderate; 3 = Moderate to Severe; 4 = Severe

**Figure 5:**
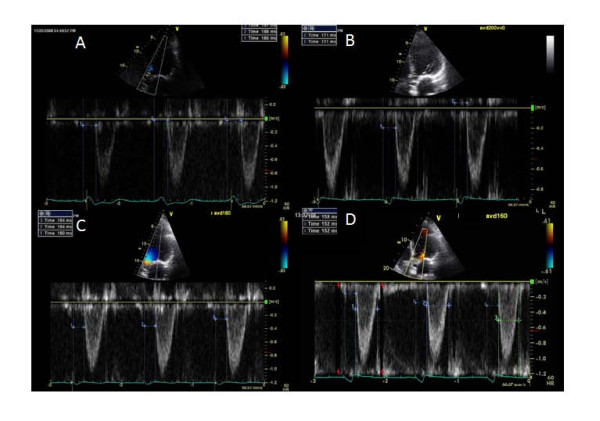
**Effect of Biventricular Pacemaker Optimization on PEP Measured by Echo Doppler**. Echo determined PEP in the same patient as in Figure 1 during native AV conduction at an AVD of 300 ms in panel A, baseline AVD of 200 ms in panel B, AVD of 180 ms in panel C and optimal AVD of 160 ms in panel C. Mean PEP was 184 ms in A and 171 ms at baseline AVD of 200 ms during biv pacing in B. AVD of 180 ms in C and optimal AVD of 160 ms in D led to shortening of PEP to 154 ms. Note difficulty in determining QRS onset in panel A.

A significant correlation was found between baseline ET and radial artery tonometry derived PEP (r = -0.44, p < 0.001) and between baseline VTI and radial artery tonometry derived PEP (r = -0.27, p < 0.001). PEP by echo could be measured in 31 out of 37 study patients. This was due to difficulty in delineating onset of QRS in all patients on the ultrasound rhythm tracing. Figure 5 shows echo PW Doppler images of LV outflow showing corresponding changes in PEP by echo during native conduction, during baseline biv pacemaker setting and during optimal AVD settings. A significant correlation was found between PEP measured by echo and PEP measured by radial artery tonometry at baseline (r = 0.44, p < 0.001) and optimal (r = 0.62, p < 0.001) pacemaker settings.

**Figure 6:**
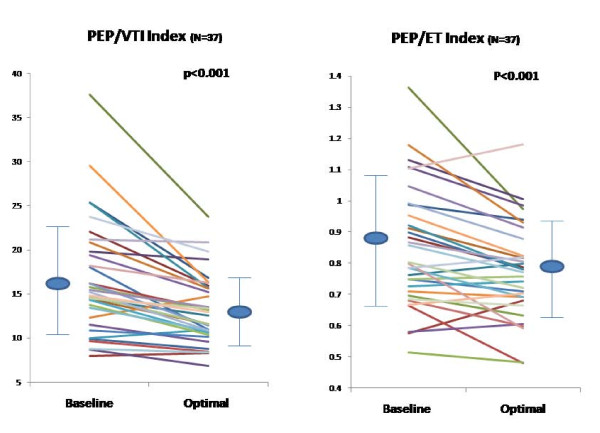
**Effect of Biventricular Pacemaker Optimization on PEP measured by Radial Artery Tonometry and Echo Doppler**. Effect of biventricular pacemaker optimization on PEP/VTI (left panel) and on PEP/ET (right panel) measured by radial artery tonometry (PEP) and by echo Doppler (VTI and ET) are shown in each study subject. PEP, pre-ejection period; VTI, velocity time integral; ET, ejection time

We used PEP/ET and PEP/VTI as a surrogate for MPI - a validated parameter for LV systolic and diastolic function. Data on PEP/VTI, PEP/ET at baseline and optimal pacemaker settings using a combination of radial artery tonometry and echo measurements is shown in Figure 6.

**Figure 7:**
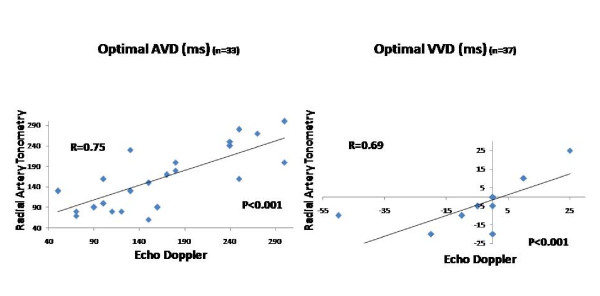
**Correlation between Echo Doppler and Radial Artery Tonometer for Optimal Pacemaker Settings**. Correlation between optimal AVD determined by radial artery tonometry and by echo Doppler (left panel) and between optimal VVD determined by radial artery tonometry and by echo Doppler (right panel). In 21 patients optimal VVD was 0 ms by both echo Doppler method and by applanation tonometry. Hence the dot at point 0 represents 21 patients instead of 1 patient. Similarly more than 1 patient (as shown in Table 2) who have identical VVD and AVD by echo and tonomtery are shown by a single data point.

Figure 7 shows the correlation between comprehensive echo Doppler and radial artery tonometry -PEP guided optimal AVD (r = 0.75, p < 0.001) and VVD (r = 0.69, p < 0.001).

Radial artery tonometry derived PEP trended to be higher (258 ± 35 ms) in those class III and IV symptoms (n = 19) compared to those with NYHA class I and II symptoms (n = 18, 235 ± 34 ms, p = 0.05). PEP was shortened to a comparable degree by pacemaker optimization in both NYHA class I-II and III-IV patients to 234 ± 42 and 236 ± 30 ms respectively.

We also evaluated baseline echocardiographic findings among those with baseline NYHA class of I and II (Gp I) versus those with NYHA class III and IV (Gp II) and the data is shown in Table 3. As shown a significant improvement in most echo Doppler parameters occurred in both groups of patients.

**Table 3:** Effect of Echocardiography Guided Biventricular Pacemaker Optimization on Cardiac Hemodynamics and Radial Artery Tonometry Derived PEP in NYHA Class I and II (Group I) vs. Class III and IV (Group II) Patients

	Parameter	Baseline	Post Opt	P value	Δ%
Group I	LV-VTI	16.8 ± 3.8	18.5 ± 4.1*	0.001	11.3
Group II	LV-VTI	15 ± 5.6	18.3 ± 6.1*	< 0.001	24†
Group I	LV-ET	298.8 ± 36.4	311.3 ± 30.9*	< 0.001	4.6
Group II	LV-ET	275.5 ± 35.3	287.5 ± 34.7*	< 0.001	4.5
Group I	FT	403.7 ± 84.2	420.8 ± 53.2	0.24	6.8
Group II	FT	440.2 ± 105.5	449 ± 100.8	0.33	2.8
Group I	MPI	0.57 ± 0.2	0.45 ± 0.15*	< 0.001	-19.6
Group II	MPI	0.58 ± 0.18	0.45 ± 0.12*	< 0.001	-19.5
Group I	MR	1.4 ± 1.1	1 ± 0.9*	0.02	-13.7
Group II	MR	1.6 ± 1.1	1.2 ± 0.9*	0.02	-22
Group I	PAP	39.5 ± 10.1	33.1 ± 9.9*	< 0.001	-17.6
Group II	PAP	52.3 ± 7.2†	42.3 ± 9*	< 0.001	-19.4
Group I	PEP	235 ± 34	234 ± 42	0.48	-0.6
Group II	PEP	258 ± 35	236 ± 30	< 0.001	-8.2†

Gp I, n = 18, Gp II, n = 19,*p < 0.05 vs. baseline; †p < 0.05 vs. group I; Δ% = Difference% = (Post Optimization-Baseline)/Baseline; LV-VTI (cm) = Left Ventricular Velocity Time Integral; ET (ms) = Left Ventricular Ejection Time; FT (ms) = Left Ventricular Filling Time; MPI = Myocardial Performance Index; MR = Mitral Regurgitation; PAP (mmHg) = Pulmonary Artery Pressure; PEP = Pre-Ejection Time (ms). MR Grade: 0 = None; 0.5 = Trace; 1 = Mild; 1.5 = Mild to Moderate; 2 = Moderate. P = 0.06 for E wave and 0.08 for DT vs. baseline.

### Follow-up

Patients were followed at 1.8 ± 1.4 months post optimization. 3 patients, one with severe ventricular ectopy requiring reversal to original pacemaker settings, 1 with recurrent hospitalizations for end stage renal disease and one wheelchair bound from multiple sclerosis were excluded. In 29 of the remaining 34 patients, NYHA class reduced from 2.5 ± 0.8 to 2 ± 0.9 (p = 0.003). NYHA improved in 18 (62%), did not change in 7 (24%) and deteriorated in 4 (14%) patients.

## Discussion

The major finding of this study is that an acute PEP shortening by radial artery tonometry occurs with optimization of AVD and VVD and correlates with improvement in hemodynamics measured by echocardiography. PEP predicted optimal VVD and optimal AVD in majority of patients. Ratio of PEP with echocardiographic LV ejection parameters - ET and VTI, corresponded to MPI assessment and shortened during pacemaker optimization. Findings from our study suggest that radial artery tonometry-PEP may be used in conjunction with echo derived ET or VTI to guide AVD optimization. The shortening of PEP was more pronounced in patients with NYHA class III and IV symptoms in whom PEP should be tested as a stand-alone method to guide pacemaker optimization.

While there was concordance between echo derived optimal AVD and radial artery tonometry derived PEP in majority of patients, some patients had significant discrepancy. This could be related to the use of comprehensive echo Doppler parameters to guide echo optimization compared to only PEP by radial artery tonometry. A randomized study comparing echo vs. PEP guided optimization and effect on acute and chronic improvement in cardiac structural and functional parameters would be required to compare the two techniques.

MPI is preload and afterload dependent and has diagnostic and prognostic power in HF patients [4,27,28]. Echocardiographic studies have shown that PEP and PEP/ET ratio can be used instead of MPI to assess cardiac performance [1,29,30]. We found PEP/VTI and PEP/ET derived from radial artery tonometry (PEP) and echo Doppler (VTI and ET) to correspond to MPI as measured by echo reflects the accuracy of PEP measured by radial artery tonometry. We have measured ET by radial artery tonometry in a prior study and showed good correlation with echo derived ET [21]. Hence PEP/ET can be derived entirely from radial artery tonometry once software modification allows simultaneous measurement of both PEP and ET. We found echocardiographic assessment of PEP less feasible due to inadequate QRS signal onset on the ultrasound monitor. Radial artery PEP was averaged over 10 beats and was a computerized measure as opposed to manual measurement of echo PEP from 3-5 Doppler tracings. Moreover onset of aortic ejection click may or may not be readily visible if the pulsed wave sample volume is placed in the desired location to measure stroke volume and it may be subject to gain artifacts and to Doppler filter settings. A higher PEP by radial artery tonometry in those with a higher NYHA class in our study group indicates that radial artery PEP is a reliable marker of LV dysfunction. Although significant acute improvement occurred in patients with NYHA I-II as well as III-IV patients, greater improvement in LV VTI occurred in those with NYHA class III and IV compared to those with NYHA class I and II and significant PEP improvement occurred only in patients with NYHA class III and IV suggesting that optimization by echo Doppler or PEP may be maximally useful in this group of patients. In contrast to echocardiographic studies, operating radial artery tonometry device is much simpler and less labor intensive and may play a potentially important role in assisting echo Doppler and other algorithms for multiple inter and intraventricular delay changes during pacemaker programming in future generation pacemakers. In particular it may have a role in determining optimal LV-RV delay and LV-LV delay in pacemakers with multiple LV and RV electrodes. Evaluation of LV performance during a single component of cardiac cycle may by itself not provide adequate evaluation of global cardiac function, and evaluation of LV performance in various systolic and diastolic phases is likely required for determination of optimal AV and VV delays. PEP was shown to correlate well with myocardial contractility provided that LV end diastolic pressure did not change significantly [3]. In this respect, we have shown that phonocardiographic S3 intensity correlates with echo Doppler measures during pacemaker optimization [19]. Radial artery PEP and better still PEP/ET may be useful in conjunction with assessment of S3 intensity for pacemaker optimization given that PEP and ET determine LV systolic performance during isovolumic and ejection systolic phase whereas S3 intensity is a marker of LV diastolic filling and diastolic pressure.

Many different echo Doppler parameters have been used in the literature for pacemaker optimization. These include mitral inflow VTI, filling time, E/A ratio, deceleration time, aortic outflow VTI, ejection duration and MR Dp/Dt. There is variability in appropriate pacemaker settings depending on which of these echo Doppler parameters is selected. It is to be noted that in almost all earlier CRT studies echo guided pacemaker optimization was used using mitral inflow or aotic outflow method. As shown in our study, comprehensive echo Doppler optimization does have a role in the management of patients - especially non responders post CRT, however it is time consuming and requires expertise. It is therefore important to find simple non invasive methods for pacemaker optimization. Radial artery tonometry technique is a simple, non invasive, fast, reproducible method of assessing PEP that can be rapidly mastered by hospital/ECG/echo staff. It eliminates operator dependent inter observer variability inherent in many echo Doppler techniques due to operator and patient related factors. Use of PEP by radial artery tonometer needs to be tested in randomized prospective studies for its value in pacemaker optimization.

## Limitations

Echo Doppler method of pacemaker optimization we describe is time intensive. The QRS width changes in response to AVD and VVD changes may influence PEP measurement independent of mechanical effects of pacemaker changes. We chose the optimal pacemaker settings based on results of echo evaluation. We did not use radial artery tonometry as the primary method of pacemaker optimization and this utility of the device needs to be tested against echocardiography in prospective randomized studies. Our patients' baseline pacemaker settings were in a wide range at baseline and not at "out of box settings". This is because patients had pacemaker programming performed by their treating physicians by using various methods and algorithms.

## Conclusion

Our findings indicate significant concordance among echocardiographic and radial artery tonometry methods in determining optimal AVD and VVD. The additive clinical impact of the PEP optimization approach over a conventional comprehensive hemodynamic assessment using echo-Doppler techniques during optimization as a stand-alone method needs to be addressed in future studies.

## Competing interests

Nima Taha, MD: Partial fellowship support from AtCor Medical.

Jing Zhang, MD, PhD: Partial fellowship support from AtCor Medical.

The sponsor did not contribute to study design, data collection, data analysis or interpretation of results and did not contribute in any way to this manuscript.

Reza Rafie, MD: None

Rupesh Ranjan, MD: None

Salima Qamruddin, MD: None

Tasneem Z Naqvi, MD: Optimization of Biventricular Pacemaker Settings - Patent Pending

## Authors' contributions

NT: Performed PEP data collection during optimization, measured echo data, performed data entry and analysis, JZ: Performed PEP data collection during optimization, measured echo data, performed echo and PEP data entry, RR: Performed data entry and assisted with data analysis, RR: Assisted with data analysis, SQ: Assisted with data analysis, TZN: Conceived of the study, performed echo guided pacemaker optimization, coordinated and supervised data analysis and entry, drafted and revised manuscript. All authors read and approved the final manuscript
